# False Impressions

**Published:** 1889-03-30

**Authors:** Caroline Fothergill

**Affiliations:** Author of "An Enthusiast," "A Voice in the Wilderness," etc.


### FALSE IMPRESSIONS.

By CAROLINE FOTHERGILL, Author of "An Enthusiast," "A Voice in the Wilderness," etc.

*(Concluded from p. 400.)*

### CHAPTER XXV.—TAKING COUNSEL.

THERE is no occasion to relate all that passed between George and his sister.

Edith had to be attended to first, and when she had recovered from a long and alarming faint, and was in bed, talking wildly and utterly unconscious of who was by her side, he told Mrs. Lester that at present the nurse might be left in charge; he wished to speak to her.

It was not a long interview; it took place in George's study, behind carefully closed doors, and no one ever knew what was said on either side. Edith's last conscious exclamation, together with other words which had fallen from her lips in the unguardedness of delirium, gave him a tolerably clear idea of what had been done by Mrs. Lester. A few stern questions, which she was too frightened to answer other than truthfully, put him in possession of the true state of the case.

He said very little, but what he did say Mrs. Lester never forgot.

"You have committed an unnatural and abominable crime," he said at last, "and it cannot yet be said if you are not also a murderess. If Edith dies, her death will lie at your door. You have committed a crime which can scarcely be atoned for."

"I did it for your good," sobbed Mrs. Lester, whose defiance had all melted away, and who was more terrified at the tragic and unexpected turn affairs had taken than she could express. "I thought you would be happier with Edith, you always seemed so fond of her, and she suited you so well. I thought if you would only prefer her, it would be for your happiness."

"You did not," he answered; "you could not. You always saw me treat Edith as a mere child, and the affection I had for her was the affection a man has for a child—a favourite little sister. You thought of no one but yourself. And had you really thought of me and done it for my sake, it would not have been less a crime. Not the holiest motives could have justified such a deed."

She made no further defence, and after a pause he went on:

"After this it would be useless to attempt to go on living together. You cannot leave the house now; your presence here is necessary. I must not leave either; but we must not meet. I can keep to my study; I cannot bear to see you, and any communication that is necessary can be held through the servants. But one thing I most emphatically forbid: you must not enter Edith's room; she cannot bear to see either of us now. She was fond of us once; now, our presence—even the mention of our names—would be more disturbing to her than anything else could be. Leave her to the nurse; your place is simply to superintend, and see that all goes right. As soon as you can be dispensed with, you must leave the house."

He turned to go, but she stopped him. He was the one being in the world whom she really loved, and his anger and estrangement were as the end of the world to her; she could not endure them.

"George," she cried, "you can't leave me like this, without one word or sign that you will ever forgive me. You must forgive me, George. I cannot live without your love."

"I can't forgive you, Frances," he said, turning once more to her, and his face showed how terribly all this had shaken him. "We are brother and sister and alone in the world; neither am I perfect, that I should condemn you in everything; but this one deed is an odious crime which I cannot forget. You have ruined an innocent and happy life, and poisoned one of the purest minds that ever existed. Edith may recover, but she will never be the same again, and it is your fault. I can't forgive."

He left the house and went out-of-doors; he had an instinctive longing for the open air, but he did not know where to go or what to do. What was the right course to take in such a labyrinth as this in which he found himself? Ought he to wait until Edith recovered—if she did recover—and then make amends by asking her to marry him? In his present mood of horror and remorse he was inclined to think that was the only thing he could do. And give up Beatrice? He rebelled passionately against that thought. He could not give her up—at least, not without her consent; and yet the thought of telling her all this loathsome tale made him feel quite sick. The thought of losing her, of having her torn from him in reparation of the evil deed of someone else, immediately after he had been blessed by knowing that her love was his, was like a nightmare: he groaned aloud. Then he felt that he must see Beatrice and tell her all. She should decide. He could abide by her decision; it would certainly be right. With a sigh of relief, he turned in the direction of York Gardens.

Beatrice was at home, and as soon as their eyes met she saw that something was wrong. She rose at once and came toward him, saying:

"George, what is the matter? You have bad news for me."

"Sit down," he said, taking her hand in his, and holding it while he spoke. "I will tell you everything; it is what I have come to do."

He told her all without reserve or palliation, although it was a difficult story to tell, and he keenly felt the humiliation of it. When he had finished, he said:

"Now tell me what I must do. I am so bewildered I can't see my way at all, and I have come to you that you may decide for me."

"You have laid a heavy responsibility upon me," she answered, "but I can tell you what I think, and together we may find out the best."

"I knew you would help me. I knew you would not fail me," he said triumphantly.

"Why, of course I should not fail you," she said, with a little laugh of happiness. "What should I be worth if I did? But you must have had some ideas yourself. Tell me the very first thing that came into your mind?"

"The very first?"

"The very first."

"It was," he answered, the dark colour rising to his cheek, "that I ought to ask your permission to break off our engagement, and ask Edith to marry me as soon as she gets better. That is the truth, Beatrice. You must forgive me for saying it, but you would have it."

She nodded, but said nothing for awhile, then she said:

"I am glad you thought that; that your first impulse was to shield her, to remember that she is a weak little thing, who must be protected."

She said no more, and at last he spoke again, urging her with gentle persistence:

"But your opinion, Beatrice? I want to know what you think."

"I think you were mistaken," she said suddenly, after another pause.

"Mistaken!" he repeated, astonished. "How do you mean? I don't understand."

"I mean it was natural you should feel that; that is, it was natural to you to feel it. But I think it was a mistaken feeling all the same. You have given me a very difficult thing to do," she went on, "to decide whether the man who is now engaged to me is not in honour bound to give me up and marry another woman, whom he does not love. I suppose if it were in a novel, the heroine would decide that that is what he ought to do, and you, Edith, and myself, would all be sacrificed to a mere sentiment. But I don't believe in useless sacrifices and suffering; there is too much real and unavoidable to be reckoned with; and I don't think that would be the best thing to do. I don't believe Edith loves you. I believe she thinks she does," she went on, noticing the startled look his face took at this bold statement, "when in reality it is only gratitude for your kindness which she feels. To me it is simply impossible that a child like she is should love a man like you; she does not know or understand you, and when she is better, she will find this out for herself. Just now you almost doubt her power of recovering from such a blow; but"—with a smile in her eyes—"that is only a man's conceit. I hope a better fate is in store for Edith than to die of imaginary love for you. I hope and believe she will recover, and when she does I believe she will have outgrown this and seen her mistake. Then she will want to forget all about it and never hear it mentioned again. That is my view of the case."

"God bless you, Beatrice," he said, when she had finished. "I believe you are right, and men are very vain; but it is your fault—you make us so. But what must I do? I can't go on as if nothing had happened."

"We must both do something," she said, becoming grave again. "For the present we must give up our engagement. I mean," she went on hastily, seeing the expression his face assumed, "not that we must give one another up, but that for the present, until Edith is better and her future settled, we must behave as though we were only friends. No one must be told of our engagement, and you must not come to see me often. People must not be allowed to think there is anything between us. But do not propose to Edith; she is a woman, or will be one after this experience, and she would know quite well what was your thought and intention in asking her to marry you. It would give her far more pain than the generosity of it would give her pleasure. You must stay at home while she is so ill, but when she is out of danger it would perhaps be better for you to go away."

"I believe you are," he said, after a fit of musing; "but it is very hard. I had intended that my next going away should be on my wedding journey."

"It is hard," she said, "but we must do it."

"I know," he said, seizing her hand, "I know it is as hard for you as for me; but you are a woman, and strong."

If Dr. Temple had been present, this admission would no doubt have made him smile, and perhaps it sent Beatrice's thoughts backward, but she let it pass, only saying:

"We are both strong and happy; she is weak and unhappy; we must make a sacrifice for her."

They sat silent for some time, then he said:

"I can scarcely believe the great happiness of knowing you love me was given only to be cancelled in this way."

"If it is worth anything, it will be all the stronger for this test," she answered

As he walked homewards, after leaving Beatrice, he met Harry Fairfax, who stopped him.

"I was coming to see you to-night," he said. "You can tell me now if you will be in."

"I shall be in," replied Murray; "but unless your business is urgent, do not come. Our house is a house of mourning, and we cannot see people for pleasure."

"I hope no disaster has befallen any of you," said Harry, in great suspense.

"My niece, Miss Lester, is very ill; we do not know if she will recover."

"Pray, tell me all about it?" cried poor Harry. "It was of her I was coming to speak to you. I asked her to marry me, and she refused, but I fancied she was not altogether indifferent to me, and I was coming up this evening to ask you to use your influence with her for me. I know how highly she values your opinion."

George grasped the young man's arm, saying:

"You asked her to marry you: when was that?"

"Immediately after you came home from Switzerland. What is the matter?" for George had stamped his foot on the ground with an exclamation unclerically strong.

"Oh, my dear fellow," he said, feeling almost desperate at this addition to his burden, "everything is the matter. Do not ask me for an explanation now; you shall hear from me as soon as possible. I wish to heaven that either you or Edith had told me of this proposal when it was made."

"I am surprised you did not hear of it," said Harry. "I spoke to her again a few days after, and she said she had spoken of it to Mrs. Lester, who had agreed with her that if she did not love me she ought not to marry me."

"She told her aunt, and her aunt encouraged her to refuse you," said George, with a positively stony expression on his face.

"So I understood," said Harry.

"Worse and worse," murmured George, as he turned to go home. "Worse and worse," he murmured again, as he thought over the whole story. "Where will Frances' sin stop? She may have blighted his life too. With much less than this on his conscience many a man has committed suicide, feeling too dishonoured to live. But women"—his old mood was strong upon him, but he remembered Beatrice, and after a momentary pause, concluded with—"are different."

Laura's observations were more cutting than sympathetic, but she approved of what George and Beatrice had done; she said it was "like them both," though how, she did not explain. She was very severe on Mrs. Lester, waxing quite eloquent in her denunciation, and winding up with—

"Suppose that girl dies!"

### Chapter XXVI.—Explanations.

Edith did not die. As Beatrice had said, there was a better fate in store for her than to die of imaginary and unrequited love for George Murray. She lay long between life and death, for her system had received a shock which might have overthrown many a stronger woman than Edith Lester. But she did finally recover, and after a long time of great weakness was able to leave her bed and lie on the couch.

All this time she had seen neither her aunt nor George, nor did she ask for them. She was entirely in the hands of her nurse, and she made no request for any other society. Her nurse was a shrewd and sensible woman, and she observed how her patient shrank and winced if ever Mrs. Lester's name was accidentally mentioned in her hearing. She drew her own conclusions, and told George what she had noticed, when she gave him her daily report. He heard her in silence, and then said—

"I am afraid it will be a long time before she can bear to see Mrs. Lester, but I should be glad if you would mention my name to her and let me know as soon as she seems inclined to see me."

This first meeting passed off better than either had expected. The cause of her present state was not mentioned; George had his old kind way, and Edith was too weak to feel all the scorching shame and pain she had anticipated. George's ease of manner, too, helped to set her at her ease, and after feeling that she ought to see him as soon as she could, but that the meeting would be full of pain and embarrassment, a miserable trial to both of them, she found herself, when the first few minutes of trembling agitation were over, really enjoying the visit. She had sufficiently recovered to feel all the monotony of sick-room existence, and the change of society was very agreeable to her. After this first visit George came every day, and as soon as she was fit for the change, carried her downstairs to the drawing-room, and gave up a good deal of time to amusing her.

By degrees she got well enough to render a change of air desirable, and here a difficulty arose. She could not go away alone, and who was to take her? Her aunt was her only female relative. Edith was, as we know, the only girl in a large family of boys; her father had neither mother nor sisters living. George could not take her, and it would be impossible to send her with no companion but a maid-servant. George took this difficulty, as all his others now, to Beatrice. He told her all about it, and she listened, and said at the end—

"If she will go with me, I will take her."

"My dear Beatrice, are you in earnest?" he asked.

"You are surprised," she said, "but I really mean it. We must meet sometime, and I should like to befriend her now, when she most needs it. I believe," she went on, looking rather embarrassed and guilty, "that she always felt a little afraid of me, but that was my fault, and I had no earthly right to behave so that she was afraid of me; I will take care that she is not afraid now. I should like to see her; ask her when you get home if she will see me. Once we have met I will arrange everything."

"You are goodness itself," he said. I can't tell you how grateful I am for your help in this matter. A man is very helpless in my position."

When he went home he took a basket of flowers, gathered by Beatrice herself, with him to smooth her path with Edith.

Edith noticed them at once.

"What lovely flowers!" she said as he came in. "Where did they come from?"

"I have brought them for you from a lady who is very anxious to come and see you if you will let her."

"Who is she?" burying her face in the basket. "She must be very kind to send me such lovely things as these."

"She is very kind, and I think you would like her very much if you knew her better. It is Miss Stanford who wants to see you, Edith."

The name was almost too much for Edith. She turned very pale, put aside the flowers, and sat trembling and shrinking in her chair. George was apparently engrossed in a magazine he had taken up, and so she could recover herself unobserved. She did so before long. It suddenly flashed upon her what her emotion might mean to George; her colour returned, and with an effort at self-control, which before her illness she would have been incapable of making, she said quietly, though not without a little tremor in her voice—

"I should be very glad to see her. Please ask her to come when it is convenient to her."

Beatrice's first visit was paid the following day, and was quite successful. Her manner was the one her working friends were accustomed to see, graceful and winning, without either dignity or reserve. Edith, her fears once overcome, fell in love with her on the spot, and when she went away, begged her to come as often as she could. Beatrice did come almost every day, and when she proposed that she and Edith should go away together, the invalid agreed to the plan with an enthusiasm which touched Miss Stanford.

They went to the sea and spent a couple of months there together. Mrs. Ledward, of whom Edith could not overcome her terror, stayed at home "to mind the house," to use her own favourite expression; but she wrote long, bright letters; and George came down frequently and brought them the Mayfield news. On the surface it seemed a very embarrassing position, and perhaps all these three people wondered sometimes how it was that things went on so smoothly and happily. Laura was intensely diverted at the thought of what she called "this triangular harmony," and was anxious to come over and be a spectator of it, but Beatrice refused her consent, on the ground that she would introduce a discordant element and must find her amusement at home.

The reason for this situation was never touched upon; no one ever spoke of the occurrences in the earlier part of the year, yet all felt that this state of things could not last for ever, and perhaps all wondered who would be the first to speak.

It was Edith who broke the silence one day after George had left them to go back to Mayfield. He and Beatrice had been for a long stretch on the sands, further than Edith could have accompanied them even had she been inclined. Beatrice had returned with glowing cheeks, and George with that light of pleasure and happiness in his eyes which Edith knew well, and had begun to connect with Beatrice. After dinner, as the dusk came on, and the two women sat alone, silent, and apparently absorbed in the books they held—in reality, each thinking intently and quite unconscious of what was contained in the printed page before her—this silence was suddenly broken by Edith, speaking with a force and character which none of those who knew her would have looked for from her.

"Miss Stanford," she asked, "when are you and Mr. Murray going to get married?"

"My dear child," said Beatrice, dropping her book in genuine amazement. "What do you mean? What has put such an idea into your head?"

"I know you were going to be married," went on Edith valiantly, though her cheeks became painfully hot. "I know you were engaged before my illness, and I know you never speak of it now, but always behave as if you were only friends. I have a feeling that you are doing this on my account."

Beatrice said nothing, and Edith went on—

"Is it so, Miss Stanford? Please tell me."

By this time Beatrice knew Edith very well, what she could bear and what she could not. There was also a new note of womanly dignity in her voice which Beatrice could not disregard, and she answered simply and truthfully—

"Yes, Edith, it is so."

"I thought so. It is very kind and considerate of you, but I don't want you to do it any longer. It makes me feel very selfish too."

"You must not let it make you feel anything of the kind," said Beatrice. "Things are going very well as they are now. I think we are all very happy."

"No," said Edith; "I am not happy. I can't bear to think that an insignificant girl like I am should be standing between two people like you and Mr. Murray. It hurts me to think of it, and it makes me feel ridiculous too. Besides, there is something else," she went on, checking Beatrice, who would have spoken. She hesitated a moment and changed colour before she could make up her mind to speak. "I don't think of Mr. Murray as I did once," she went on at last. "I have thought about it a great deal, and I see now how silly I have been, but—but it was not altogether my fault. I only wanted to tell you that it won't hurt me at all when Mr. Murray gets married, and if we were at the beginning of last summer again, I would act very differently."

It is needless to tell all that Beatrice said in reply. That which interested her most was Edith's last admission, and with a little tact and management she got from her all the story of Harry Fairfax's attachment and proposal. Edith confessed that the favour with which she had even then regarded him had now strengthened into a feeling which formed the main object of her life. She thought of very little else, and was now regretting her refusal with a strength of which Beatrice would scarcely have believed her capable.

"It is all over now," said Edith mournfully, when her story was finished, "and I suppose I shall have to be an old maid. I am sure he will never ask me again when I have treated him so badly, and I shall never care for anyone else."

But Beatrice laughed at her and made light of her fears. She said she did not at all believe that Harry's feelings had changed. If his attachment was serious, of which she felt no doubt whatever, he would certainly speak again; only Edith must have patience, and not lose heart because of a little delay. She said she knew that Harry had called every day during Edith's illness, and that once, when they expected she was going to die, he had remained in the house all night, although the next day was Sunday and he had to take full duty. This last seemed to particularly touch Edith, as Beatrice, knowing her, had expected it would. The floodgates of confidence being opened, Edith revealed many things to Miss Stanford as they sat together in the dusk. Amongst other things she confessed the fear which had haunted her during the first part of her long convalescence, that George would think it necessary to try to repair the mischief which had been done by asking her to marry him.

"I was horribly afraid," she said. "Every time he spoke I thought he was going to say that, and if he had done, I think I should have died with shame; and I knew then, too, that I did not really care for him in that way, but had only thought I did. But as time went on and he never said anything, but was only very kind, I grew less afraid; and when you came to see me I knew it was all right, and that he would never remind me how silly I had been."

"What should you have said if he had asked you?" enquired Beatrice, not without curiosity.

"That was the worst of it," said Edith with solemnity. "I should have felt obliged to say "Yes," and I should not have wanted to"

The result of this conversation was, that after Edith had gone to bed, Beatrice wrote a long letter to George, and that when George came again a few days afterwards he brought Harry Fairfax with him, and asked him if he would potter about with Edith, while he and Miss Stanford took a long walk together.

Edith never regretted the answer she gave to Harry Fairfax on the cliff that afternoon, and when they were married from York Gardens three months later, she was able to return George's farewell kiss without the least change of colour or one heart-beat which was not for her husband. She was perfectly happy in her country rectory, and made an ideal country rector's wife, with her husband and children, and numerous absorbing interests. When, some years after her marriage, her aunt succumbed to her last illness, a lengthy and tedious affair, requiring great skill and patience in nursing, it was Edith who bore the burden of it all, and was with her to the last.

Beatrice and George were married too; Dr. Temple was best man, and Mrs. Ledward gave away the bride.

THE END.

### FINANCIAL POSITION OF FRIENDLY SOCIETIES.

THE financial statements for the past year have just been issued by the Ancient Order of Foresters and the Manchester Unity of Oddfellows, the two great affiliated societies, whose members exceed a million in number. The total accumulated capital of the Foresters on the 31st of December last was £3,670,114, of which £3,109,922 belonged to court, sick, and funeral funds, and £369,751 to district funeral funds. These figures show an addition of £167,931 to the reserve capital during the year, although £456,798 was paid away for sick benefits, and £104,140 for funeral payments on the death of members. The income last year was £701,660, of which £588,746 was from members' subscriptions, and £105,802 from interest on invested capital. The members to whom these funds belonged numbered 599,435. The Manchester Unity, which received returns from lodges with 553,352 members on the same date, possessed accumulated funds amounting to £6,806,736. Of this £6,121,395 belonged to the sick and funeral funds of 3,578 lodges, and £176,284 to district funeral funds, and £341,335 to widows and orphans' funds. The increase during the year was £222,507, and £535,989 was paid during 1887 for sick benefits, and £208,723 as funeral and other payments on the death of members. The income last year was £666,712, of which £650,296 was derived from members' subscriptions, and £231,747 from invested capital. These figures show the two societies paid £1,305,650 last year in benefits, and added to capital another £390,438, and their total reserve capital is £10,477,450.

